# Prevalence and predictors of gender-based violence among Wolkite University female students, southwest Ethiopia, 2021: Cross-sectional study

**DOI:** 10.3389/frph.2023.978808

**Published:** 2023-02-13

**Authors:** Haile Workye, Zebene Mekonnen, Wesen Wedaje, Aregash Sitot

**Affiliations:** ^1^ Department of Nursing, College of Health Science, Injibara University, Injibara, Ethiopia; ^2^ Department of Nursing, College of Medicine and Health Science, Wolkite University, Wolkite, Ethiopia; ^3^ Department of Midwifery, College of Health Science, Mekelle University, Mekelle, Ethiopia

**Keywords:** gender, physical, sexual, Wolkite, Ethiopia

## Abstract

**Background:**

Gender-based violence is an important topic that needs to be taken into account when identifying gender-related gaps and disadvantages that a person might face because of their gender. Violence against women could result in psychological and physical adverse outcomes. Therefore, this study aims to assess the prevalence and predictors of gender-based violence among female students at Wolkite University, southwest Ethiopia, 2021.

**Method:**

An institutional-based cross-sectional study was conducted among 393 female students and the students were selected by using a systematic sampling method. Data were checked for completeness and entered into EpiData version 3.1 and then exported to SPSS version 23 for further analysis. Binary and multivariable logistic regressions were employed to determine the prevalence and predictors of gender-based violence. The adjusted odds ratio (AOR) with its 95% confidence interval (CI) at a *p*-value of ≤0.05 was used to check statistical association.

**Result:**

In this study, the overall prevalence of gender-based violence among female students was 46.2%. The prevalence of physical violence and sexual violence was 56.1% and 47.0%, respectively. Factors that were significantly associated with gender-based violence among female university students were: being a 2nd-year student or having a lower educational level [AOR = 2.56 (95% CI, 1.06–6.17)], being married or living with a male partner [AOR = 3.35 (95% CI, 1.07–10.5], having a father with no formal education [AOR = 15.46 (95% CI, 5.204–45.39)], having a drinking habit [AOR = 2.53 (95% CI, 1.21–6.30)] and not being able to freely discuss issues with their families [AOR = 2.48 (95% CI, 1.27–4.84)]

**Conclusions and recommendations:**

The result of this study showed that more than one-third of the participants were exposed to gender-based violence. Thus, gender-based violence is an important topic deserving of more consideration; further investigations are important to decrease gender-based violence among university students.

## 1. Background

Gender-based violence (GBV) is an important topic that needs to be taken into account when identifying gender-related gaps and disadvantages that a person might face because of their gender (1, 2). GBV is “violence involving men and women, in which the woman is usually the victim, and which is derived from unequal power relationships between men and women” (3). Gender-based power differentials between men and women around the world place women at risk for multiple forms of violence, whether occurring in public or private life with short or long-term consequences for the survivors' health and well-being (4).

Women and girls around the world are more likely to experience GBV at the hands of someone they know, which can often make it harder to report abuse or seek medical, psychological, and legal support due to familial or social restrictions on their autonomy to make personal health and legal decisions, or due to social stigma and victim blaming (1). Gender-based violence is an endemic problem in most communities worldwide and a well-known public concern that can lead to physical, sexual, emotional, or financial abuse (1, 2, 5). Violence against women can result in psychological and physical adverse outcomes, such as stress, anxiety, depression, unsafe abortions, unwanted pregnancies, and sexually transmitted infections (2, 6).

Globally, 27% of partnered women of reproductive age (15–49 years old) are estimated to have experienced sexual intimate partner violence at least once in their lifetime (7). In developing countries, the prevalence of violence has been grossly underreported (8). The prevalence of gender-based violence is still high in Sub-Saharan Africa and greater than 50% in Ethiopia, Nigeria, Kenya, Uganda, and South Africa (2). The overall prevalence of gender-based violence in educational institutions of Sub- Saharan Africa ranged from 42.3% in Nigeria to 67.7% in Ethiopia (7).

GBV against female students is a salient issue and women who attend university campuses are the main victims (9). Currently in Ethiopia, the prevalence of GBV is still alarmingly high in educational institutions (10). A systematic review and meta-analysis study showed that the prevalence of sexual violence among female higher education students in Ethiopia was 49.4% (11). Another systematic review and meta-analysis study in Ethiopia showed that the prevalence of workplace sexual violence among females was 22%, which is lower than the gender-based violence occurring in higher education (12). Overall lifetime prevalence of gender-based violence among female students was 67.7% in Debremarkos (13), 58.3% in East Gojjam (3), 68.2% in Southeast, AletaWondo (14), and 63.2% in Woliata Sodo (6). Different factors contribute to the occurrence of gender-based violence among female students, such as low academic performance, lack of sexual and reproductive health education, and poor family involvement. These factors increased significantly the risk of gender-based violence (15). Additionally, female students who were living alone in a rented house or had a partner (husband or boyfriend) also had higher probabilities of suffering gender-based violence (16). Other factors as the absence of family discussion on women's reproductive health and related personal issues, if a person witnessed GBV family abuse, and if there was alcohol abuse also had a significant association with gender-based violence (17). Ethiopia is a multicultural country but most of the local cultures are structured patriarchally with a notion of male dominance over women. This has a great impact on the roots of gender-based violence, including for female university students. It is crucial that all concerned bodies are involved to modify this patriarchal belief (18).

However, problems are abundant and most studies were conducted within the high school setting, while this study emphasized specifically the problem of gender-based violence in higher education institutions. To assess the prevalence and predictors of GBV and provide strategies and strengthen existing preventive strategies, female students who took part in this study were physically apart from their families.

## 2. Methodology

### 2.1. Study area and period

Wolkite University (WKU) is one of the third-generation higher institutions in Ethiopia and was established in 2012. It is located in the Southern Nation Nationalities People Regional State (SNNPRS), Gurage Zone, 158 km in the southwest direction from Addis Ababa, the capital of Ethiopia. It is situated at Gubreye Sub-city, 10 km from Wolkite town, the capital of Guraghe Zone, on Gubrye to Butajira road. WKU has eight colleges: College of Social Science and Humanities, College of Agriculture Science, College of Natural and Computational Sciences, College of Computing and Informatics, College of Engineering and Technology, College of Health Science, College of Business and Economics, and College of Education and Behavioural Science. It also has two schools, the School of Law and the School of Medicine, and more than 46 departments. It provides education for undergraduate and postgraduate students, and has a total of 10,000 students. The study was conducted in April 2021.

### 2.2. Study design

An institutional-based cross-sectional study was conducted.

### 2.3. Study population and source of population

#### 2.3.1. Source population

All female students of Wolkite University registered in 2020.

#### 2.3.2. Study population

All sampled female students of Wolkite University.

### 2.4. Inclusion

The study population included all female students registered for the year 2020 in the colleges and who attended at least one semester.

### 2.5. Sample size determination

The sample size of the study was determined by using a single population proportion formula. This study considered the prevalence of experiencing any form of gender-based violence in their lifetime, which was 63.2% among female students in Wolaita Sodo (6), with 5% marginal error and 95% CI (*α* = 0.05). By using this calculation formula, the final sample size was 393.

### 2.6. Sampling technique and procedure

First, three colleges were randomly selected from a total of seven colleges. Then, proportional allocation was done for each college and a stratified sampling method was used to select the female students based on their year level. Then, simple random sampling was used to collect the data from the sampled students.

### 2.7. Data collection procedure

Data were collected by using a structured self-administered questionnaire. The questionnaire was adapted from different literature and had four parts: socio-demographic, family history, sexual history, and behavioral attributes toward gender-based violence. It was considered gender-based violence if the study participants had suffered sexual or physical violence. Data were collected by ten data collectors who had bachelor's degrees and two instructors supervised the data collection procedure. Two days of training were given to data collectors and supervisors so that there was a common understanding and awareness of the context of each question in the questionnaire. The pre-test was done on 5% of the study's participants in Wolkite College before the actual date of data collection and correction was made based on the findings.

### 2.8. Variables

#### 2.8.1. Dependent variable

• Gender-Based Violence (GBV)

#### 2.8.3. Independent variable

• **Socio-demographic characteristics**: such as age, residence, education level, religion, marital status, etc.
• **Family history:** family living nearby, marital status and educational status of the family, witness of parental violence, etc.
• **Sexual history**: sexual activity, age at first sexual intercourse, willingness at first sexual intercourse and reasons for lack of willingness, number of sexual partners, etc.
• **Behavioral attributes**: such as drinking alcohol, chewing chat, smoking, and their frequencies.

### 2.9. Data processing and analysis

First, the collected data were coded, checked, and entered into EpiData version 3.1 and then exported into SPSS version 23 for analysis. The result of the uni-variable analysis (descriptive results) was presented as frequencies and percentages. The bivariable analysis was carried out to determine the association of each independent variable with the outcome variable. Variables that had an association with the outcome variable at a *p*-value ≤ 0.2 were selected for multivariable analysis. The multivariable analysis was performed in the logistic regression to control the confounding factors. The Hosmer Lemeshow test was done to test the model's goodness of fit. Odds ratios with 95% CI were used to show the strength and direction of the associations. Finally, a variable with a *p*-value of less than 0.05 was considered statistically significant.

### 2.10. Ethical consideration

Before data collection, ethical clearance was obtained from Wolkite University College of Medicine and Health Sciences, Department of Nursing, and dispatched to each college. The study participants received adequate information about the study, confidentiality, and their rights during data collection, and written informed consent was taken from the participants before data collection was started.

## 3. Results

### 3.1. Socio-demographic characteristics

Overall, a total of 353 female students participated with a response rate of 100%. Most of the respondents, 292 of them (82.7%), were between the age of 18–24 years, 122 (34.6%) had Orthodox religious beliefs, and 248 (70.3%) lived in urban centers before joining the university. In this study, 332 participants (94.1%) were living on campus while 21 (5.9%) were living outside the campus. Regarding their marital status, 52 (14.7%) were married, and 315 (30.8%) had boyfriends (Table 1).

**Table 1:** Socio-demographic characteristics among female Wolkite University students, southwest Ethiopia, 2021 (*n* = 353).

Variable	Category	Frequency (*n*)	Percentage (%)
Age (years)	18–24	292	82.7
	25–34	56	15.9
	35–45	5	1.4
Religion	Orthodox	122	34.6
	Muslim	99	28.0
	Protestant	106	30.0
	Catholic	26	7.4
Residence before joining the university	SNNPR	100	28.3
	Addis Ababa	76	21.5
	Oromia	98	27.8
	Amhara	37	10.5
	Tigray	28	7.9
	Others	14	4.0
Residence before joining the university	Urban	248	70.3
	Rural	105	29.7
Current living situation	On-campus	332	94.1
	Outside campus	21	5.9
Educational level	Year 2	100	28.3
	Year 3	117	33.1
	Year 4 and above	136	38.5
Have boyfriend	Yes	52	14.7
	No	301	85.3

### 3.2. Family history of the respondents

Among the study participants, 288 (81.6%) were living with their parents, 79 (22.4%) had fathers who received no formal education, 100 (28.1%) had mothers who received no formal education, 148 (41.9%) had been eyewitnesses of their mothers being beaten by their husbands/male partners (Table 2).

**Table 2:** Family history of respondents among female university students, southwest Ethiopia, 2021 (*n* = 353).

Variable	Category	Frequency (*n*)	Percentage (%)
Parents living together	Yes	288	81.6
	No	65	18.4
Father's education	No formal education	79	22.4
	Formal education	274	77.6
Mother's education	No formal education	100	28.3
	Formal education	253	71.7
Mothers beaten by their husbands/male partners	Yes	148	41.9
	No	205	58.1

### 3.3. Behavioral attributes of the respondents

In this study, the participants who had a history of chewing chat and smoking cigarettes/tobacco were 27.8% and 21.2%, respectively. Half of them, 183 participants (51.8%), drank alcohol at least once in their life and 261 (73.9%) had been drunk at least once in their life. The frequency of behavioral attributes was as follows: 22 (6.3%) chewed chat once/twice a week, 25 (9.9%) smoked once/twice a week, 65 (18.5%) drank once/twice a week, and 9 (2.5) used drugs once/twice a week (Table 3).

**Table 3:** Behavioral attributes among female university students, southwest Ethiopia, 2021 (*n* = 353).

Variable	Category	Frequency (*n*)	Percentage (%)
Chewing chat	Yes	98	27.8
	No	255	72.2
Chewing frequency	Every day	35	9.9
	Once/twice a week	22	6.3
	1–3 times a month	21	6.0
	Occasionally < 1 month	5	1.4
	Others	14	4.0
Smoking	Yes	75	21.2
	No	278	78.8
Smoking frequency	Every day	14	4.0
	Once/twice a week	25	9.9
	1–3 times a month	7	2.0
	Occasionally < 1 month	5	1.4
	Others	14	4.0
Has been drunk once before	Yes	183	51.8
	No	170	48.2
Drinking alcohol frequency	Every day	7	2.0
	Once/twice a week	65	18.5
	1–3 times a month	39	11.1
	Occasionally < 1 month	57	16.2
	Others	14	4.0
Since joining university	Yes	232	65.7
	No	29	8.2
Started drinking in the year the study was conducted	Yes	162	45.9
	No	35	9.9
Drug use (cocaine)	Yes	35	9.9
	No	318	90.1
Drug use frequency	Once /twice a week	9	2.5
	1–3 times a month	14	4.0
	Occasionally < 1 month	12	3.4

### 3.4. Sexual experiences of the respondents

Of the total respondents, 239 (67.7%) had a history of sexual experience, 207 (58.6%) had their first sexual intercourse between the ages of 18–24 years, and 83 (23.5%) had more than four sexual partners until the time of the study. Of the participants, 183 (51.8%) had sexual intercourse unwillingly due to various reasons: 54 (15.3%) had drunk alcohol, 64 (18.1%) had received false promises, 73 (20.7%) received financial support and 48 (13.6%) had sex in exchange of passing marks in an exam. Among the participants, 57 (16.1%) had freely discussed with their family members about reproductive health issues (Table 4).

**Table 4:** Sexual experiences among female university students, southwest Ethiopia, 2021 (*n* = 353).

Variable	Category	Frequency (*n*)	Percentage (%)
Have you ever had sexual intercourse	Yes	239	67.7
	No	114	32.3
Age at first sexual intercourse	18–24 years	207	58.6
	≥25 years	32	9.1
How old was the person with whom you had your first sexual intercourse	Do not know	178	50.4
	18–24 years	46	13.0
	≥25 years	15	4.2
Were you willing when you first had sex	Have not had sex	114	32.3
	Yes	56	15.9
	No	183	51.8
Reason for sexual intercourse unwillingness	No	114	32.3
	Drunken alcohol	54	15.3
	False promise	64	18.1
	Financial support	73	20.7
	To pass an exam	48	13.6
How many sexual partners have you had until now	None	114	32.3
	One	94	26.6
	Two	29	8.2
	Three	33	9.3
	Four or more	83	23.5
Able to freely discuss issues with family member	Yes	57	16.1
	No	296	83.9

### 3.5. Offenders and consequences of physical and sexual violence

Among the participants who had experienced physical and sexual violence, 75 of them (21.2%) reported that physical violence was committed by teachers and 56 (15.9%) reported that sexual violence was committed by family members. In the study, 85 (24.1%) of the victims of physical violence reported having poor academic performance, 26 (4.5%) claimed the sexual violence led them to be considered as “disgusting people”, 24 (6.8%) of the victims of physical violence had withdrawn from or failed school, and 21 (5.9%) had suffered a temporary body injury due to physical violence. Different physical, psychological, and social consequences were reported as a result of sexual violence. These were as follows: 84 participants (23.8%) developed an unusual vaginal discharge, 53 (15%) became pregnant, 33 (9.9%) had abortions, 71 (20.1%) were exposed to fear, 60 (17.0%) felt hopelessness, 51 (14.4%) had poor academic achievement/failed from school, and 51(14.4%) withdrew from school (Table 5).

**Table 5:** Offenders and consequences of physical and sexual violence among female university students, southwest Ethiopia, 2021 (*n* = 353).

Status	Category	Frequency (*n*)	Percentage (%)
Physical violence offenders	None	169	47.9
	Boyfriend/husband	19	5.4
	Family members	7	2.0
	Other (non-relative)	9	2.5
	Teacher	75	21.2
	Student	60	17.0
	Others (police officers, drivers…)	14	4.0
Consequence of physical violence	No consequence	178	50.4
	Poor school achievement	85	24.1
	Withdrawal from school	24	6.8
	Causing disgust in other people	26	4.5
	Temporariy body injury	21	5.9
	Sustained disability	8	2.3
	Others	21	5.9
Sexual violence offenders	None	173	49.0
	Boyfriend/husband	7	2.0
	Family member	56	15.9
	Other (non-relatives)	26	7.4
	Teacher	32	9.1
	Students	30	8.5
	Others	29	8.2
	None	173	49.0
Physical consequences of sexual violence	None	155	43.9
	Unusual vaginal discharge	84	23.8
	Swelling/injury around genitalia	19	5.4
	Pregnancy	53	15.0
	Abortion	33	9.9
	Others	7	2.0
Psychological consequences of sexual violence	None		
	Self-blame	21	5.9
	Fear	71	20.1
	Anxiety	26	7.4
	Hopelessness	60	17.0
	Suicidal thoughts	7	2.0
	Others	7	2.0
Social consequences of sexual violence	None	154	43.6
	Poor achievement/failure in school	51	14.4
	Withdrawal from school	51	14.4
	Rejection from family	23	6.5
	Rejection from friends/peers	30	8.5
	Alcohol dependency/abuse	15	4.2
	Sexual dependency/abuse	15	4.2
	Having multiple sexual partners	7	2.0
	Others	7	2.0

### 3.6. Prevalence of gender-based violence and physical and sexual violence among female students

The prevalence of physical violence among female university students was 198 (56.1%) before joining the university, 184 (52.1%) since joining the university, and 154 (43.6%) in the year the study was conducted; and the sexual violence suffered by female university students was 175 (49.5%) before joining the university, 123 (34.8%) since joining the university, and 99 (28.0%) in the year the study was conducted (Table 6).

**Table 6:** Prevalence of physical and sexual violence before joining the university, since joining the university, and in the year the study was conducted; among female Wolkite University students, 2021.

Variables		Before joining university		Since joining university		In the year the study was conducted (2020)	
		Frequency (*n*)	Percentage (%)	Frequency (*n*)	Percentage (%)	Frequency (*n*)	Percentage (%)
Physical Violence	Yes	198	56.1	184	52.1	154	43.6
	No	155	43.9	169	47.9	199	56.4
Sexual Violence	Yes	175	49.6	123	34.8	99	28.0
	No	178	50.4	230	65.2	254	72

### 3.7. Overall physical violence and sexual violence suffered by the students during their lifetime

**Figure 1:**
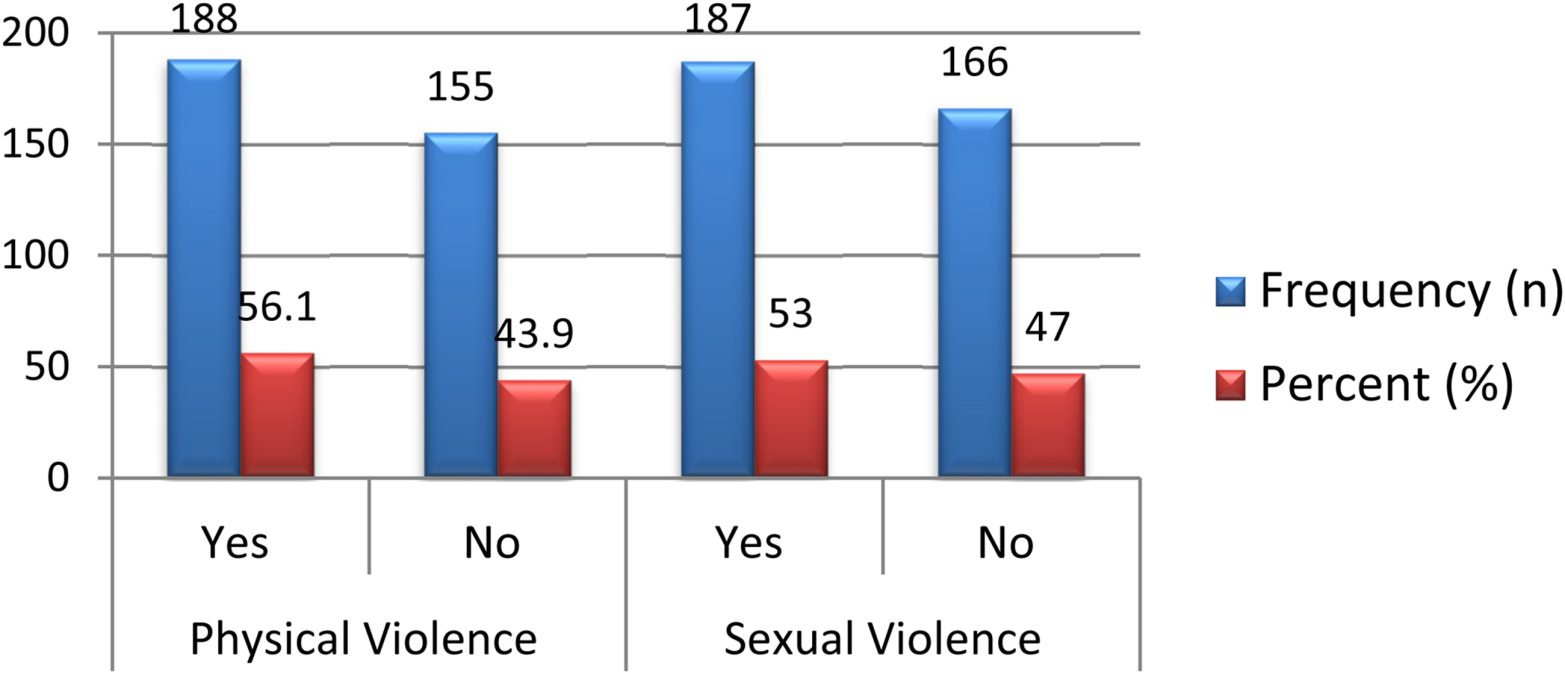
Prevalence of physical and sexual violence among female university students in their life time, southwest Ethiopia, 2021.

The prevalence of physical and sexual violence among female Wolkite University students was 188 (56.1%) with 95% CI [51%–61.5%] and 187 (53.0%) with 95% CI [47.9–58.4], respectively (Figure 1).

### 3.8. Gender-based violence suffered by female students in their lifetime

**Figure 2:**
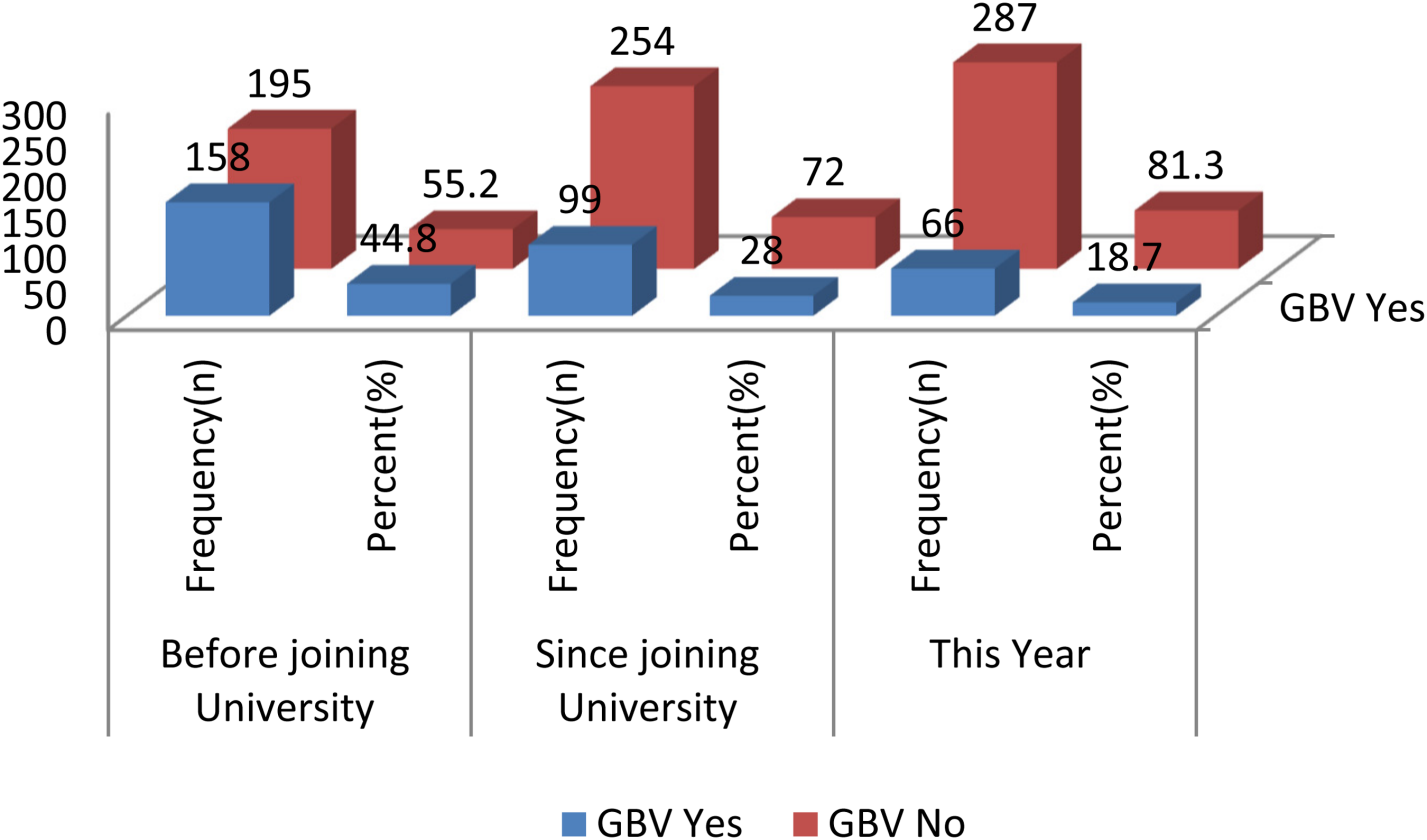
Prevalence of GBV before joining University, since joining University and this year among female University students, southwest Ethiopia, 2021.

The GBV suffered by the female students before joining the university was 158 (44.8%), 99 (28%) since joining the university, and 66 (18.7%) in the year the study was conducted (Figure 2).

### 3.9. The overall GBV suffered by female students during their lifetime

**Figure 3:**
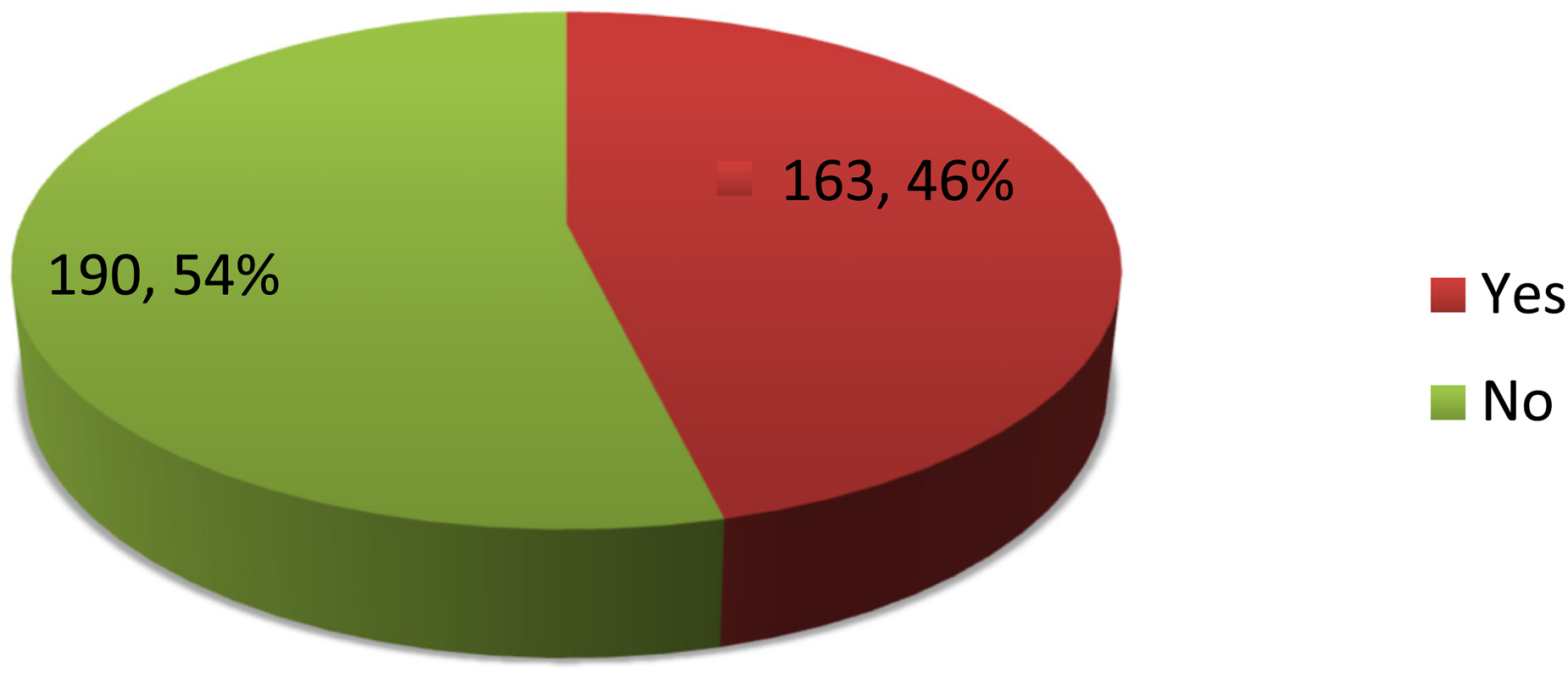
Overall prevalence of GBV among female Wolkite University students, southwest Ethiopia, 2021.

The overall prevalence of gender-based violence among female Wolkite University students throughout their lives was 163 (46.2%) with 95% CI (40.8–51.3) (Figure 3).

### 3.10. Factors associated with gender-based violence

In the multivariable logistic regression, the respondent's educational level, their father's educational level, their drinking habit, and their freedom to discuss reproductive health issues with family members were statistically significant factors with a 5% level of significance. The female students who were in their 2nd year or below were three times more likely to be exposed to gender-based violence [AOR =  2.56 (95% CI, 1.06–6.17)]. Those married or living with a male partner were 3.35 times more likely to face gender-based violence [AOR = 3.35 (95% CI, 1.07–10.5)]. The female students whose fathers had no formal education were 15.46 times more likely to face GBV [AOR = 15.46 (95% CI, 5.204–45.39)]. Those with alcohol-drinking habits were 2.53 times more likely to be the victim of GBV [AOR = 2.53 (95% CI, 1.21–6.30)], and students who were not able to freely discuss with family members about their reproductive health were 2.48 times more likely to face GBV [AOR = 2.48 (95% CI, 1.27–4.84)] (Table 7).

**Table 7:** Factors significantly associated with gender-based violence among female university students, southwest Ethiopia, 2021*.*

Item	Category	GBV		COR (95% CI)	AOR (95% CI)	*p*-value
		Yes = *n* (%)	No = *n* (%)			
Educational level	2nd year and below	39(39.0%)	61 (61.0%)	0.97 (0.57–1.65)	2.56 (1.06–6.17)	0.037*
	3rd year	72 (61.5%)	45 (38.5%)	0.387 (0.23–0.64)	0.03 (0.01–0.12)	<0.001*
	4th year and above	52 (38.2%)	84 (61.8%)	1	1	
Fathers education	No formal education	23 (29.1%)	46 (70.9%)	2.54 (1.48–4.37)	15.46 (5.204–45.39)	<0.001*
	Formal education	140 (51.1%)	134 (48.9%)	1		
Been drunk once before	Yes	97 (53.1%)	86 (46.9%)	2.29 (1.25 5.68)	2.53 (1.21–6.30)	0.016*
	No	57 (33.6%)	113 (66.4%)	1	1	
Able to freely discuss issues with family members	Yes	73 (57.5%)	54 (42.5%)	1	1	
	No	90 (39.8%)	136 (60.2%)	5.66 (2.87–11.14)	2.48 (1.27–4.84)	0.008*

## 4. Discussion

In this study, the overall prevalence of gender-based violence (GBV) among female students was 46.2%, which was similar to the 52.8% reported in the study conducted in sub-Saharan Africa (7), but lower than the findings from other studies, which were as follows: the Aleta Wondo study found a prevalence of 68.2% (13), the Gojam study found a prevalence of 58.3% (3), and the Wolayita Sodo study reported a prevalence of 63.2%. These differences might be due to different study periods as the reproductive health services and the awareness of women's reproductive health and rights within the communities shift and increase.

However, this finding is higher than the reported in a US study that found a gender-based prevalence of 25% (19). This variation might be due to the difference in socio-economic status and difference in health care delivery systems.

In this study, the prevalence of physical violence was 56.1%, which is similar to the result of 56.14% reported in the study conducted in Aleta Wondo (14) and 56.3% reported in Wolayita Sodo (6). In this study, sexual violence was 47.0%, which is higher than the figure reported in Aleta Wondo (26.3%) (14) and Wolayita Sodo (37.2%) (6). This variation is due to different study periods and different socio-economic statuses.

Female students who were in their 2nd year or below were 2.56 times more likely to experience gender-based violence than female students who were in their 4th year or above. This study was supported by the study conducted in Wolayita Sodo (6). The possible reason for the discrepancy in gender-based violence according to academic level could be that as the grade level increases, the awareness and knowledge about sexual and reproductive health issues also increase. Also, students are more likely to create a safer environment for themselves by challenging their peers to reflect on their behavior and speaking up when someone crosses the line, or by enlisting the help of others if they don't feel safe. The study participants whose fathers had no formal education were 15.46 times more likely to be exposed to gender-based violence than participants whose fathers had formal education. The possible explanation might be that fathers who have no formal education may have less of a close connection with their daughters and avoid discussing their reproductive rights. The study participants who had drinking habits were 2.53 times more exposed to GBV than participants who had no drinking habits. This finding was supported by the studies conducted in Wolayita Sodo, Aleta Wondo, and Mombasa, Kenya (6, 14, 20), respectively. The reason might be that heavy drinking may contribute to increased exposure to violence, including increased risks of intimate partner violence and sexual assault. The study participants who had no male partner were 3.35 times more likely to be exposed to gender-based violence than participants who had a male partner. The possible explanation may be the fact that in Ethiopia there are harmful social norms that contribute to gender inequality. For example, there is a patriarchal mentality that a man can override a woman's choices over her body and that women's bodies should always be available to men. Female students who were not able to freely discuss issues with family members were 2.48 times more likely to suffer gender-based violence than participants who were free to discuss assorted topics with family members. The possible reason might be that family acts as a bridge connecting individuals and the larger society, so being able to freely discuss various issues with family is important to promote and protect women's rights to control and decide freely over matters related to their sexuality, including sexual and reproductive health, and family-planning possibilities.

## 5. Conclusion and recommendation

### 5.1. Conclusion

The finding of this study showed that the prevalence of gender-based violence was very high among Wolkite University female students and the factors that contributed to gender-based violence included drinking habits, low levels of education, having fathers with no formal education, and not being able to freely discuss issues with family.

### 5.2. Recommendations

Based on the major findings of the study, the following specific areas of intervention are suggested for better feature mitigation of gender-based violence in the study area.

᠅ Raising awareness among adolescents and families.
᠅ Social and cultural influences on gender-based violence need to be changed to the treatment and reassurance of survivors of sexual assault.
᠅ Raising awareness among both female and male higher education students.
᠅ Use peer-to-peer communication to develop skills on how to prevent violence in individuals and friends.
᠅ Universities and concerned bodies should work hard and promote female students' awareness about their legal rights concerning sexual violence.
᠅ A community-based survey is recommended to assess the magnitude of sexual violence among housemaids not attending an educational institution.

## Data Availability

The original contributions presented in the study are included in the article/Supplementary Materials, further inquiries can be directed to the corresponding author.
